# Sex-Specific Relationship Between Parathyroid Hormone and Platelet Indices in Phenotypes of Heart Failure—Results From the MyoVasc Study

**DOI:** 10.3389/fcvm.2021.682521

**Published:** 2021-06-16

**Authors:** Bianca Dahlen, Felix Müller, Sven-Oliver Tröbs, Marc William Heidorn, Andreas Schulz, Natalie Arnold, M. Iris Hermanns, Sören Schwuchow-Thonke, Jürgen H. Prochaska, Tommaso Gori, Hugo ten Cate, Karl J. Lackner, Thomas Münzel, Philipp S. Wild, Marina Panova-Noeva

**Affiliations:** ^1^Preventive Cardiology and Preventive Medicine, Department of Cardiology, University Medical Center of the Johannes Gutenberg-University Mainz, Mainz, Germany; ^2^DZHK (German Center for Cardiovascular Research), Partner Site Rhine Main, Mainz, Germany; ^3^Clinical Epidemiology and Systems Medicine, Center for Thrombosis and Hemostasis, University Medical Center of the Johannes Gutenberg-University Mainz, Mainz, Germany; ^4^Cardiology I, Department of Cardiology, University Medical Center of the Johannes Gutenberg-University Mainz, Mainz, Germany; ^5^Laboratory for Clinical Thrombosis and Hemostasis, Department of Internal Medicine, Cardiovascular Research Institute Maastricht, Maastricht University Medical Center, Maastricht, Netherlands; ^6^Institute for Clinical Chemistry and Laboratory Medicine, University Medical Center of the Johannes Gutenberg-University Mainz, Mainz, Germany

**Keywords:** heart failure, MPV, platelet count, parathyroid hormone, heart failure with preserved ejection fraction, heart failure with reduced ejection fraction

## Abstract

**Background:** Heart failure (HF) is a multifactorial syndrome with pathophysiological complexities still not fully understood. Higher mean platelet volume (MPV), a potential marker of platelet activation, and high concentrations of parathyroid hormone (PTH) have been implicated in the pathogenesis of HF.

**Aim:** This study aims to investigate sex-specifically the association between PTH concentrations and platelet indices in phenotypes of HF.

**Methods and Results:** PTH and platelet indices (MPV and platelet count) were available in 1,896 participants from the MyoVasc study in Mainz, Germany. Multivariable linear regression models, adjusted for age, sex, season, vitamin D status, cardiovascular risk factors, comorbidities, estimated glomerular filtration rate, and medication, were used to assess the associations between platelet indices and PTH. The results showed distinct sex-specific associations between PTH and platelet indices. A positive association between PTH and MPV was found in females with symptomatic HF with reduced ejection fraction (HFrEF) only [β = 0.60 (0.19; 1.00)]. Platelet count was inversely associated with PTH in male HFrEF individuals [β = −7.6 (−15; −0.30)] and in both males and females with HF with preserved ejection fraction (HFpEF).

**Conclusion:** This study reports differential, sex-specific relationships between PTH and platelet indices in HF individuals independent of vitamin D status and clinical profile. Particularly in phenotypes of symptomatic HF, distinct associations were observed, suggesting a sex-specific mechanism involved in the interaction between PTH and platelets.

## Introduction

Heart failure (HF) is one of the most common cardiovascular diseases (CVDs) accounting for substantial morbidity and mortality worldwide, with increasing incidence and prevalence especially among the elderly (1). As a heterogeneous condition, HF syndrome comprises predominantly two phenotypes (1). HF with preserved ejection fraction (HFpEF) is more frequent in females with cardiovascular comorbidities, whereas males with history of ischemic heart disease suffer more often from HF with reduced ejection fraction (HFrEF) (2, 3).

Recently, elevated parathyroid hormone (PTH) concentrations have been associated with all-cause and cardiovascular mortality in HF patients, suggesting a potential role for PTH in the pathogenesis and progression of HF (4, 5). PTH is physiologically released at low calcium concentrations to stimulate the synthesis of the active form of vitamin D, Calcitriol, which in turn suppresses PTH release as a negative feedback regulation of calcium homeostasis (6). Besides calcium concentrations, plasma PTH concentrations were also modulated by age and renal function (4, 7). Higher concentrations of PTH have been associated with advanced stages of HF according to categories of the New York Heart Association (NYHA) (8, 9), reduced left ventricular ejection fraction (LVEF) (8), and elevated brain natriuretic peptide (BNP) or N-terminal propeptide of BNP (NT-proBNP) (10–12). Different pathways have been proposed for the interaction of PTH with the heart. As a stimulator of hypertrophy, arrhythmia, and inflammation, PTH directly drives cardiomyocyte necrosis and thus accelerates the severity of HF (8, 11). In addition, PTH indirectly exacerbates HF by the activation of the renin–angiotensin–aldosterone system (RAAS), a key element of HF pathophysiology (13).

Platelet activation has been associated with traditional cardiovascular risk factors (CVRFs) and CVD including the HF syndrome (14, 15). Higher mean platelet volume (MPV), a potential marker of platelet activation, was reported in individuals with arterial hypertension, obesity, dyslipidemia, and diabetes mellitus (16). We have recently reported on sex-specific determinants of MPV in the general population with age, smoking, arterial hypertension, and high blood glucose concentrations linked with higher MPV in males, whereas oral contraceptives and menstrual bleeding were associated with higher MPV in females (14).

Platelet activation including higher MPV, increased whole blood aggregation tendency, and higher platelet-bound and soluble P-selectin has been associated with HF syndrome (14, 15). Positive associations between MPV and PTH were described in individuals with primary hyperparathyroidism and end-stage renal failure patients (17, 18). In addition, an experimental study showed an important enhancing effect of the PTH-related protein, a protein initially isolated from hypercalcemia-associated tumors, on agonist-induced platelet activation and aggregation (19). Individuals with coronary artery disease presenting with higher PTH concentrations showed increased ADP-mediated platelet aggregation and suboptimal response to clopidogrel, despite receiving a dual antiplatelet therapy (7).

The relation between platelet function and PTH plasma concentration has been poorly explored in individuals with HF. This analysis aimed to investigate sex-specifically the associations between PTH concentrations and the platelet indices, platelet count, and MPV, across phenotypes of HF in individuals enrolled in the MyoVasc study.

## Methods

### Analysis Sample

As a large prospective cohort study at the University Medical Center of the Johannes Gutenberg-University Mainz in Germany, the MyoVasc Study was primarily conceptualized to investigate the development and progression of HF and its interaction with vascular disease (20). The study included 3,289 participants aged from 35 to 84 years. All subjects underwent an extensive, standardized clinical and laboratory investigation including sampling of biomaterials for biobanking at the MyoVasc study center. Platelet count, MPV, and PTH were available in the first 2,000 participants enrolled in the MyoVasc study at their baseline examination between January 2013 and January 2016. The assessment of CVRFs, comorbidities, and medication as well as echocardiography of cardiac structure and function are described in the Supplementary Material (Part A). Written informed consent was obtained from all study participants prior to entering the study. The study complies with the principles outlined in the Declaration of Helsinki, Good Clinical Practice and Good Epidemiological Practice. An approval from the responsible ethics committee [reference number 837.319.12 (8420-F)] and data safety commissioner was obtained in 2012, before study initiation. The MyoVasc study was registered at http://clinicaltrials.gov (identifier: NCT04064450).

### Definition of HF Phenotypes

Based on measurement of LVEF following a standardized echocardiographic assessment (Supplementary Material Part A), subjects with LVEF ≥ 50% were defined as having preserved ejection fraction (EF) and those with LVEF < 50% as having reduced EF, independent of presence of HF symptoms. Individuals with symptomatic HF (i.e., HF, stage C or D according to AHA) were further categorized according to LVEF into (i) HF with preserved ejection fraction (HFpEF) with LVEF ≥ 50%, (ii) HF with reduced ejection fraction (HFrEF) with LVEF ≤ 40%, and (iii) HFpEF borderline with LVEF in the range of 41–49% according to the ACCF/AHA Guideline for the Management of Heart Failure (21).

### Laboratory Assessment

Venous blood sampling was performed for laboratory markers of the present analysis by using tripotassium ethylenediaminetetraacetic acid (K3-EDTA) tubes. Platelet count (10^9^/L) and MPV (femtoliter, fl) were automatically determined on an ADVIA 120 Hematology System (Siemens, Erlangen, Germany) within 30 to 90 min after blood withdrawal in the Central laboratory of the Institute for Clinical Chemistry and Laboratory Medicine, University Medical Center Mainz, Germany. PTH was measured in pg/ml by an immunoassay with an automated chemiluminescence analyzer (Liaison XL, DiaSorin, Saluggia, Italy) in the Biomolecular Laboratory of the Clinical Epidemiology and Systems Medicine, Center for Thrombosis and Hemostasis, University Medical Center Mainz, Germany.

### Data Management and Statistical Analysis

Statistical analysis was performed after data quality control including a review for correctness, completeness, representativeness, accuracy, and plausibility performed by the data management unit. Baseline characteristics of the analysis sample were presented according to phenotype of cardiac function. Normally distributed values were described by mean and standard deviation (SD), non-normally distributed variables were described by median and interquartile range. Associations between platelet indices (i.e., MPV and platelet count) and PTH were presented per phenotype of cardiac function by linear regression models, adjusted for the following variables in stepwise extended models: (i) age, sex, season, and vitamin D status; (ii) plus additionally with CVRFs (diabetes mellitus, arterial hypertension, smoking, dyslipidemia, obesity, and family history of myocardial infarction and stroke) and estimated glomerular filtration rate (eGFR); (iii) plus comorbidities subsuming CVD, venous thromboembolism (VTE), chronic obstructive pulmonary disease (COPD), cancer, and arthritis; and (iv) plus additionally medication (vitamin D supplements, calcium supplements, diuretics, beta-blockers, calcium channel blockers, RAAS antagonists, antiplatelet agents, antilipemic drugs, anti-inflammatory and rheumatic drugs, glucocorticoids, corticosteroids, antibacterial drugs, and immunosuppressant drugs). The subgroup analysis in males was conducted with adjustment for the same covariates as the whole analysis sample, whereas in females, it was additionally adjusted for oral contraceptives, hormone replacement therapy, and menstrual bleeding in the full model.

Because of the explorative character of the analysis, a significance threshold for *p*-values was not defined and *p*-values were interpreted as a continuous measure of statistical evidence. All statistical analyses were performed using R, version 3.6.0 (R Foundation for Statistical Computing, Vienna, Austria; http://www.r-project.org).

## Results

### Baseline Characteristics of the Analysis Sample

**Figure 1:**
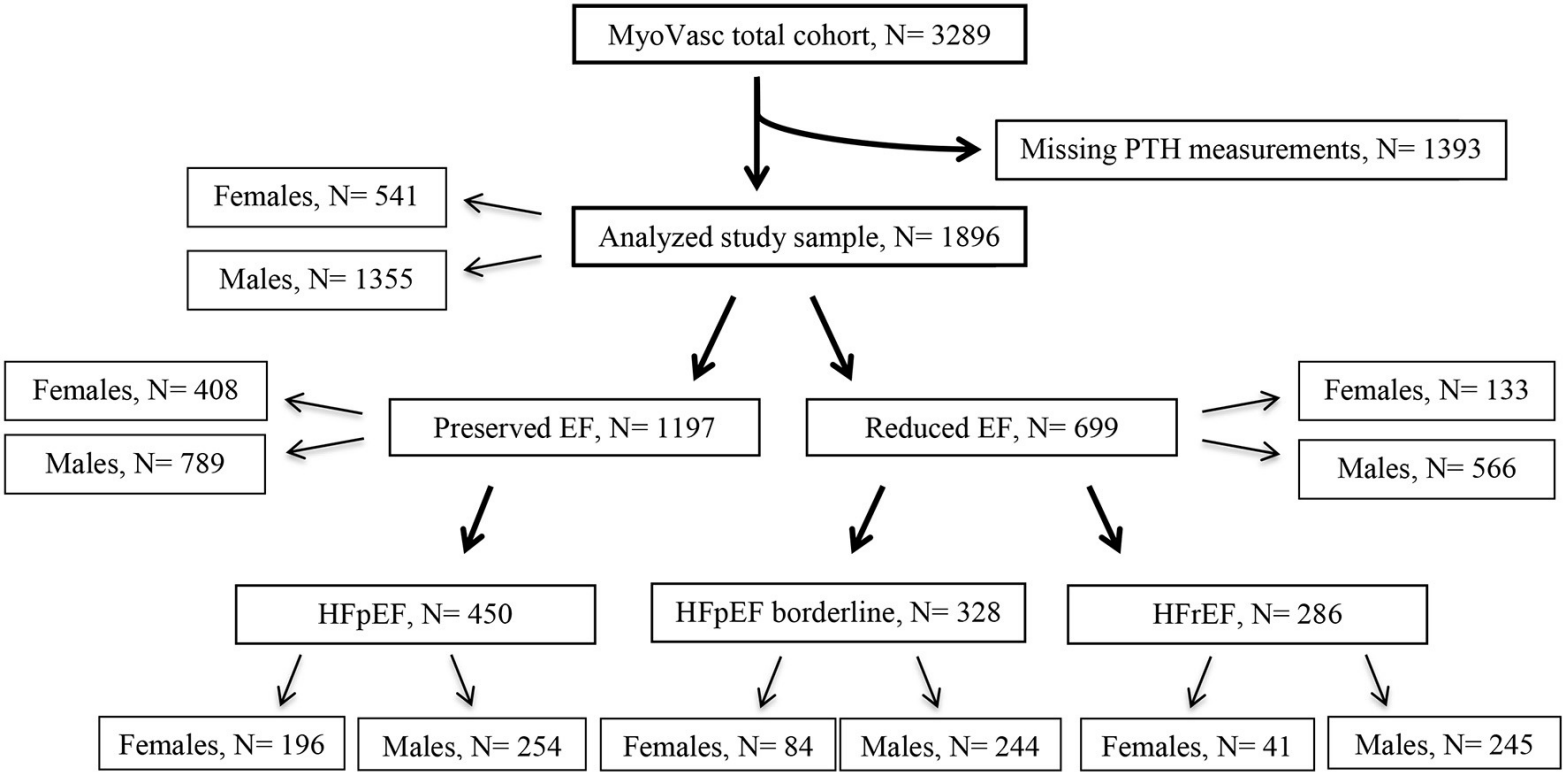
Derivation of the analysis sample. Flow chart presenting the derivation of the analysis sample based on measurements of PTH and ejection fraction. *N*, number of individuals; PTH, parathyroid hormone; EF, ejection fraction; HFpEF, heart failure with preserved ejection fraction; HFpEF borderline, heart failure with LVEF 41 to 49%; HFrEF, heart failure with reduced ejection fraction.

After exclusion of individuals with missing data on PTH, 1,896 subjects were available for analysis (Figure 1). Baseline characteristics of the individuals in the analysis sample are reported in Table 1 according to phenotype of cardiac function. Based on EF and irrespective of presence of symptoms, 1,197 individuals were characterized with preserved EF and 699 individuals with reduced EF. Symptomatic HF was present in 1,064 (56.1%) individuals, of whom 42.3% (450) had HFpEF, 30.8% (328) HFpEF borderline, and 26.9% (286) HFrEF. More than 80% of individuals with reduced EF and HFrEF were males with a higher frequency of smokers, dyslipidemia, coronary artery disease, and history of myocardial infarction compared to individuals with preserved EF and HFpEF, respectively. In the subgroup with preserved EF and HFpEF, there were more females comparatively to the other phenotypes, but overall still more males. Individuals with preserved EF and HFpEF had more often arterial hypertension and a history of VTE compared to other phenotypes. Subjects with HFpEF borderline had the lowest proportion of diabetes mellitus (28.7 vs. 32.7% in HFpEF and 33.9% in HFrEF).

**Table 1:** Baseline characteristics according to phenotype of cardiac function (*N*= 1,896).

	**Phenotype of cardiac function**				
	**Preserved EF (*N* = 1,197)**	**Reduced EF (*N* = 699)**	**HFpEF (*N* = 450)**	**HFpEF borderline (*N* = 328)**	**HFrEF (*N* = 286)**
Age [years]	67.2 ± 9.4	65.6 ± 10.6	70.7 ± 8.2	66.2 ± 10.6	65.6 ± 10.6
Sex (women)	34.1% (408)	19.0% (133)	43.6% (196)	25.6% (84)	14.3% (41)
**CVRFs**					
Arterial hypertension	84.0% (1,006)	74.8% (523)	86.2% (388)	78.7% (258)	75.9% (217)
Diabetes mellitus	25.1% (300)	30.3% (212)	32.7% (147)	28.7% (94)	33.9% (97)
Smoking	10.5% (126)	17.3% (121)	9.1% (41)	17.1% (56)	18.9% (54)
Obesity	34.4% (412)	35.2% (246)	38.7% (174)	38.4% (126)	36.0% (103)
Dyslipidemia	79.1% (947)	84.4% (590)	78.2% (352)	84.5% (277)	86.0% (246)
FH of MI/stroke	24.3% (290)	27.0% (189)	23.3% (105)	27.7% (91)	29.0% (83)
**Comorbidities**					
History of MI	30.7% (368)	39.3% (275)	28.4% (128)	37.5% (123)	43.7% (125)
History of Stroke	10.3% (123)	10.4% (73)	10.4% (47)	11.9% (39)	10.8% (31)
CAD	50.9% (609)	56.2% (393)	49.8% (224)	57.6% (189)	57.3% (164)
AF	26.9% (322)	37.6% (263)	36.9% (166)	38.7% (127)	40.2% (115)
History of VTE	11.2% (134)	9.3% (65)	14.2% (64)	10.1% (33)	8.7% (25)
History of Cancer	16.8% (201)	17.6% (123)	19.6% (88)	18.3% (60)	16.8% (48)
**Echocardiographic parameters**					
EF [%]	58.3 ± 5.2	39.5 ± 8.1	58.1 ± 5.4	45.2 ± 2.8	31.5 ± 5.9
E/E'	8.57 (6.65/11.30)	10.17 (7.26/14.47)	11.16 (8.76/14.62)	9.22 (7.05/12.74)	12.40 (8.34/18.02)
**Lab parameters**					
MPV [fl]	8.22 ± 0.86	8.36 ± 0.93	8.25 ± 0.84	8.31 ± 0.89	8.43 ± 0.99
Platelet count [10^9^/L]	219 (182/260)	208 (173/251)	218 (179/261)	213 (177/260)	206 (170/244)
PTH [pg/ml]	30.0 (23.0/38.4)	34.0 (26.3/46.7)	32.1 (23.6/42.3)	32.7 (24.8/45.3)	38.6 (29.1/52.1)
eGFR [ml/min/1.73 m^2^]	76.44 ± 18.62	71.98 ± 21.50	69.80 ± 19.42	73.80 ± 20.54	66.36 ± 22.36
**Medication**					
Vitamin D supplements (A11CC)	8.7% (104)	5.9% (41)	8.7% (39)	5.5% (18)	6.6% (19)
Calcium supplements (A12A)	1.9% (23)	1.9% (13)	3.1% (14)	2.7% (9)	1.0% (3)
Antihypertensiva (C02)	4.4% (53)	2.1% (15)	5.8% (26)	2.7% (9)	1.4% (4)
Diuretics (C03)	28.5% (341)	66.1% (462)	43.6% (196)	60.1% (197)	86.4% (247)
Beta-blockers (C07)	69.0% (826)	79.4% (555)	75.8% (341)	79.6% (261)	84.6% (242)
Calcium channel blockers (C08)	25.4% (304)	14.2% (99)	32.2% (145)	19.8% (65)	8.4% (24)
Renin–Angiotensin–Aldosterone system antagonsists (C09)	78.6% (941)	81.3% (568)	80.2% (361)	85.4% (280)	86.0% (246)
Lipid-modifying agents (C10)	60.4% (723)	60.9% (426)	58.9% (265)	64.3.% (211)	60.1% (172)
Antithrombotic agents (B01A)	80.6% (965)	85.6% (598)	85.1% (383)	88.4% (290)	86.0% (246)

*Presented are baseline clinical characteristics, echocardiographic and laboratory parameters, including intake of medications according to cardiac function phenotype in 1,896 subjects. EF, ejection fraction; HFpEF, heart failure with preserved ejection fraction (EF ≥ 50%); HFpEF borderline, heart failure with ejection fraction of 41%−49%; HFrEF, heart failure with reduced ejection fraction (EF ≤ 40%); CVRFs, cardiovascular risk factors; FH, family history; MI, myocardial infarction; CAD, coronary artery disease; AF, atrial fibrillation, PAD, peripheral artery disease, COPD, chronic obstructive pulmonary disease; VTE, venous thromboembolism; CKD, chronic kidney disease; EF, ejection fraction; MPV, mean platelet volume; PTH, parathyroid hormone*.

Similarly to the clinical profile, differences between HF phenotypes were also evident in laboratory parameters: individuals with reduced EF presented with higher MPV and PTH concentrations, but lower platelet count as well as worse renal function (determined by eGFR), compared to individuals with preserved EF. Within the subsample with symptomatic HF, highest MPV and PTH and lowest platelet count and worst renal function were observed in individuals with HFrEF.

Individuals with preserved EF and particularly subjects with HFpEF were more frequently taking vitamin D supplements, antihypertensives, and calcium channel blockers compared to those with reduced EF, HFpEF borderline, and HFrEF. Intake of diuretics, beta-blockers, and antithrombotic agents were more often reported for subjects with reduced EF, HFpEF borderline, and HFrEF.

Pearson's correlation sex-specific analysis between PTH levels and age and according to HF phenotype showed a weak correlation in both males and females across different HF phenotypes as presented in Supplementary Table 1.

### Association Between MPV and PTH

**Figure 2:**
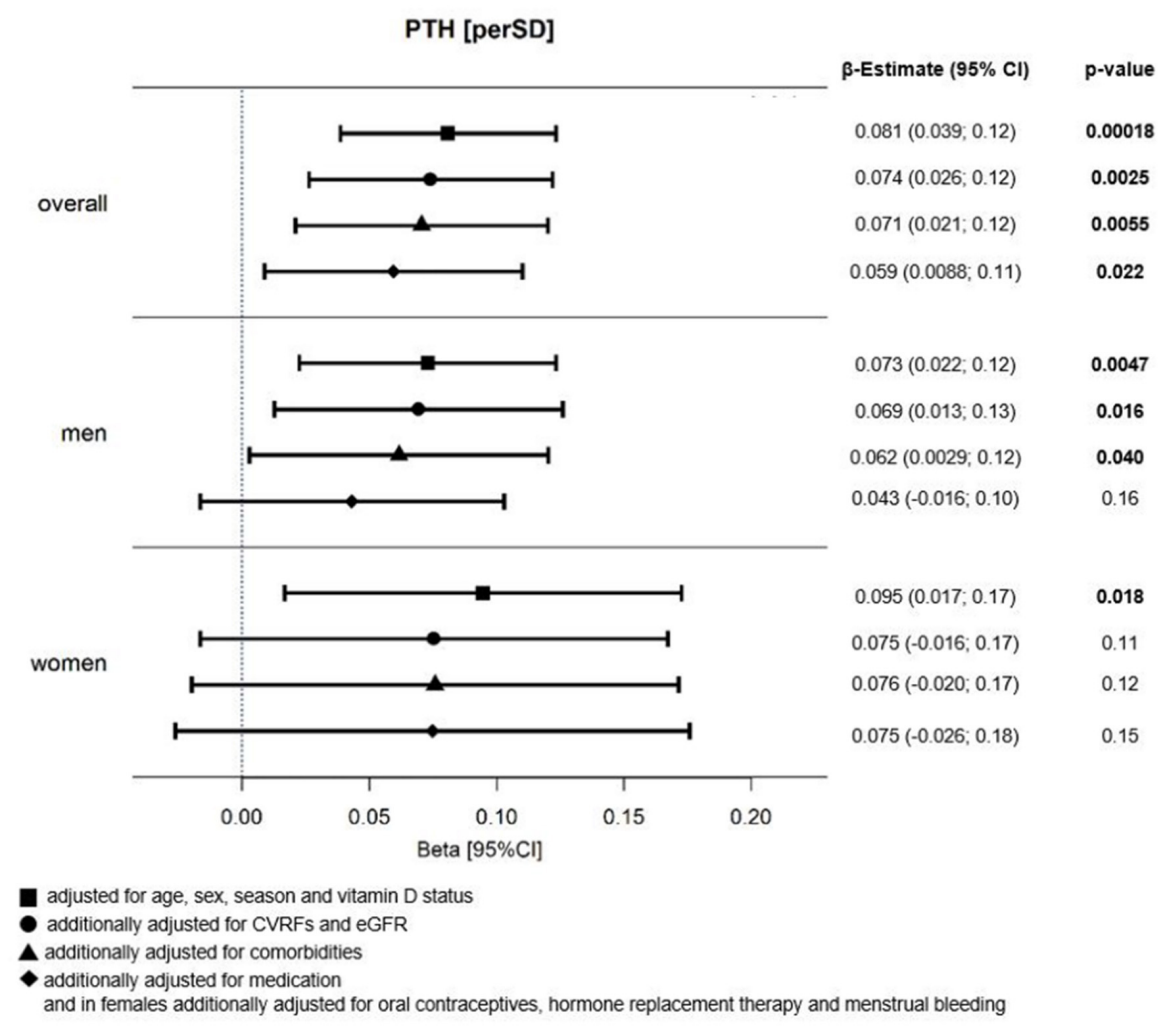
Relation between MPV and PTH in HF individuals. Forest Plot of beta (β)-estimates with 95% confidence intervals (CIs) for the relation between MPV and PTH in all HF individuals and stratified by sex. *N* = 1,861; thereof *N* = 532 females and *N* = 1,329 males; adjustment for sex only in overall analysis sample; MPV, mean platelet volume; PTH, parathyroid hormone; CVRFs, cardiovascular risk factors; eGFR, estimated glomerular filtration rate.

In the whole sample, a positive association between MPV and SD change of PTH with beta estimate (β) = 0.081 (95% confidence interval: 0.039; 0.12) was observed after adjustment for age, sex, season, and vitamin D status, which corresponded in males to β = 0.073 (0.022; 0.12) and in females to β = 0.095 (0.017; 0.17). Results from a linear regression model for MPV are presented in Figure 2. Further adjustment for CVRFs plus eGFR, comorbidities, and medication did not significantly change this association in the whole sample. A sex-specific analysis showed a mildly stronger association between MPV and PTH in females compared to males. The analysis stratified for cardiac function showed important sex-specific differences between phenotypes (Table 2): there was a positive association between MPV and PTH independent of age, season, and vitamin D status in individuals with preserved EF [β = 0.078 (0.020; 0.14)], which was only present in male participants [β = 0.11 (0.034; 0.18)], whereas in reduced EF and HFrEF, MPV and PTH were associated in females only [β_reducedEF_ = 0.21 (0.043; 0.37); β_HFrEF_ = 0.36 (0.063; 0.67)] after the same adjustment. For HFpEF borderline, a weak association was only found in women. Interestingly, the strongest and most robust association was found in females in HFrEF, where it remained relevant even after adjustment for CVRFs and comorbidities.

**Table 2:** Relation between MPV and PTH according to cardiac function in a sex-specific analysis.

		**MPV**							
		**Adjusted for age, sex^a^, season, vitamin D status^b^**		**Additionally adjusted for CVRFs and eGFR**		**Additionally adjusted for comorbidities**		**Additionally adjusted for medication^c^**	
	***N***	**β-estimate (95% CI)**	***P*-value**	**β-estimate (95% CI)**	***P*-value**	**β-estimate (95% CI)**	***P*-value**	**β-estimate (95% CI)**	***P*-value**
Preserved EF	1,174	0.078 (0.020; 0.14)	**0.0086**	0.077 (0.013; 0.14)	**0.019**	0.060 (−0.0066; 0.13)	0.078	0.043 (−0.025; 0.11)	0.21
Females	401	0.014 (−0.078; 0.11)	0.77	−0.023 (−0.13; 0.084)	0.67	−0.039 (−0.15; 0.073)	0.50	−0.046 (−0.17; 0.073)	0.45
Males	773	0.11 (0.034; 0.18)	**0.0044**	0.12 (0.037; 0.20)	**0.0045**	0.10 (0.016; 0.18)	**0.020**	0.074 (−0.011; 0.16)	0.090
Reduced EF	687	0.067 (0.0012; 0.13)	**0.046**	0.064 (−0.011; 0.14)	0.093	0.073 (−0.0051; 0.15)	0.067	0.071 (−0.0089;0.15)	0.082
Females	131	0.21 (0.043; 0.37)	**0.015**	0.26 (0.050; 0.46)	**0.016**	0.27 (0.055; 0.49)	**0.016**	0.25 (0.029; 0.47)	**0.029**
Males	556	0.030 (−0.041; 0.10)	0.41	0.021 (−0.061; 0.10)	0.62	0.025 (−0.061; 0.11)	0.57	0.023 (−0.065; 0.11)	0.61
HFpEF	442	0.075 (−0.0046; 0.15)	0.065	0.049 (−0.040; 0.14)	0.28	0.031 (−0.063; 0.12)	0.52	0.019 (−0.076; 0.11)	0.69
Females	191	0.033 (−0.086; 0.15)	0.59	−0.0011 (−0.15; 0.14)	0.99	−0.018 (−0.17; 0.13)	0.81	n.a.	n.a.
Males	251	0.10 (−0.0087; 0.21)	0.073	0.076 (−0.045; 0.20)	0.22	0.056 (−0.073; 0.18)	0.40	0.028 (−0.10; 0.16)	0.68
HFpEF borderline	324	0.046 (−0.051; 0.14)	0.35	0.040 (−0.070; 0.15)	0.48	0.038 (−0.076; 0.15)	0.51	0.029 (−0.090; 0.15)	0.63
Females	82	0.22 (0.0076; 0.44)	**0.046**	0.22 (−0.066; 0.50)	0.14	0.21 (−0.095; 0.51)	0.18	0.10 (−0.30; 0.50)	0.62
Males	242	−0.028 (−0.14; 0.080)	0.61	−0.036 (−0.16; 0.087)	0.57	−0.044 (−0.17; 0.084)	0.50	−0.051 (−0.19; 0.087)	0.47
HFrEF	279	0.11 (0.0062; 0.21)	**0.038**	0.11 (−0.0033; 0.23)	0.058	0.14 (0.016; 0.26)	**0.028**	0.12 (−0.0055; 0.24)	0.062
Females	41	0.36 (0.063; 0.67)	**0.024**	0.59 (0.19; 0.99)	**0.0071**	0.60 (0.19; 1.0)	**0.0089**	n.a.	n.a.
Males	238	0.072 (−0.036; 0.18)	0.19	0.059 (−0.065; 0.18)	0.35	0.083 (−0.049; 0.22)	0.22	0.079 (−0.056; 0.22)	0.25

*Multivariable linear regression analysis with MPV as dependent variable and PTH as independent variable in phenotypes of cardiac function and sex-specific. Results are presented as beta (β)-estimates for change per 1 standard deviation in PTH. MPV, mean platelet volume; PTH, parathyroid hormone; N, number of individuals; eGFR, estimated glomerular filtration rate; EF, ejection fraction; HFpEF, heart failure with preserved ejection fraction; HFpEF borderline, heart failure with ejection fraction of 41–49%; HFrEF, heart failure with reduced ejection fraction; n.a., not available due to low sample size*

a *Sex-adjustment only in overall analysis sample;*

b *Vitamin D status was determined by concentrations of Calcifediol and Calcitriol;*

c *In females additionally adjusted for oral contraceptives, hormone replacement therapy and menstrual bleeding. P-value < 0.05 were highlighted in bold*.

### Association Between Platelet Count and PTH

**Figure 3:**
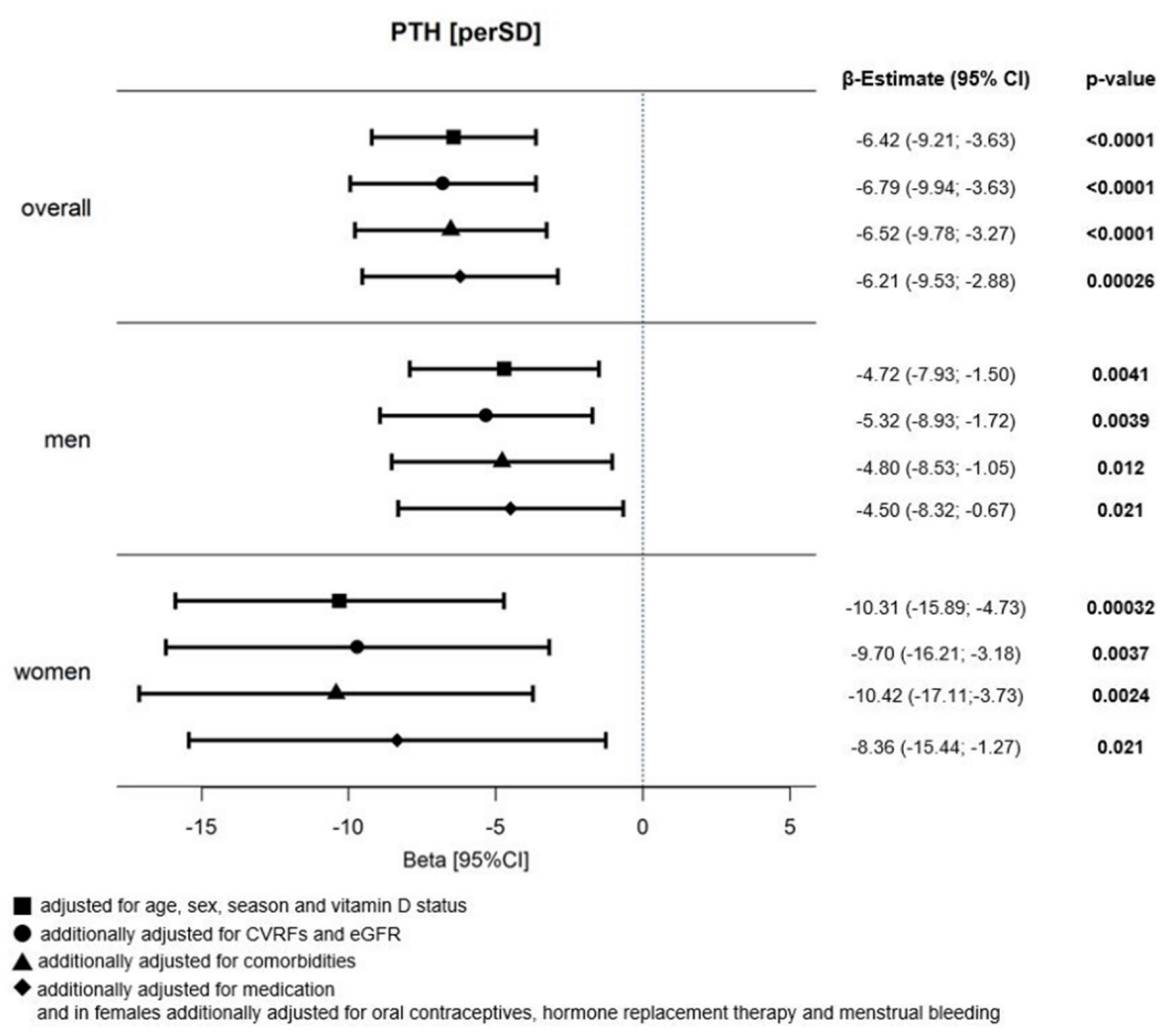
Relation between platelet count and PTH in HF individuals. Forest Plot of beta (β)-estimates with 95% confidence intervals (CIs) for the relation between platelet count and PTH in all HF individuals and stratified by sex. *N* (total analysis sample) = 1,860, thereof *N* = 532 females and *N* = 1,328 males; adjustment for sex only in overall analysis sample; PTH, parathyroid hormone; CVRFs, cardiovascular risk factors; eGFR, estimated glomerular filtration rate.

Results of the multivariable analysis for platelet count showed a strong inverse association per SD of PTH independent of age, sex, season, and vitamin D status with β = −6.42 (−9.21; −3.63), which remained after further adjustment for CVRFs and eGFR [β = −6.79 (−9.94; −3.63)], comorbidities [β = −6.52 (−9.78; −3.27)], and medication [β = −6.21 (−9.53; −2.88)] in the whole analysis sample (Figure 3). This reciprocal association was observed in males and females independent of all potential confounders, but with higher estimates in females then in men [β_females_ = −8.36 (−15.44; −1.27) vs. β_males_ = −4.50 (−8.32; −0.67)].

The analysis according to cardiac phenotypes, as presented in Table 3, showed relevant associations between platelet count and PTH in both individuals with preserved [β = −6.7 (−11; −2.0)] and reduced ejection fraction [β = −5.6 (−11; −0.77)]. In HFpEF, the largest effect estimates for an inverse association between PTH and platelet count were found, and these remained robust after adjustment for age, sex, season, and vitamin D status [β = −9.5 (−15; −3.6)], but also further adjustment for CVRFs and eGFR [β = −9.9 (−17; −3.2)], comorbidities [β = −9.4 (−16; −2.4)], and medication [β = −8.9 (−16; −1.7)]. The sex-specific analysis in this phenotype showed stronger associations in females than in males. Differently, in HFrEF, the inverse association between PTH and platelet count was only found in male individuals and present independent of all considered confounders [β = −7.6 (−15; −0.30)]. No associations were observed between platelet count and PTH in individuals with HFpEF borderline.

**Table 3:** Relation between platelet count and PTH according to cardiac function in a sex-specific analysis.

		**Platelet count**							
		**Adjusted for age, sex^a^, season, vitamin D status^b^**		**Additionally adjusted for CVRFs and eGFR**		**Additionally adjusted for comorbidities**		**Additionally adjusted for medication^c^**	
	***N***	**β-estimate (95% CI)**	***P*-value**	**β-estimate (95% CI)**	***P*-value**	**β-estimate (95% CI)**	***P*-value**	**β-estimate (95% CI)**	***P*-value**
Preserved EF	1,173	−7.0 (−11; −3.0)	**0.00071**	−7.2 (−12; −2.8)	**0.0015**	−6.9 (−11; −2.3)	**0.0032**	−6.7 (−11;−2.0)	**0.0052**
Females	401	−11 (−18; −3.3)	**0.0046**	−10 (−19; −1.9)	**0.016**	−10 (−19; −1.7)	**0.019**	−8.0 (−17; 1.1)	0.084
Males	772	−5.0 (−9.8; −0.17)	**0.043**	−5.2 (−10; 0.060)	0.053	−4.8 (−10; 0.64)	0.084	−4.4 (−10; 1.1)	0.12
Reduced EF	687	−4.4 (−8.3; −0.41)	**0.031**	−5.7 (−10; −1.1)	**0.015**	−5.3 (−10; −0.52)	**0.030**	−5.6 (−11; −0.77)	**0.024**
Females	131	−5.9 (−14; 2.4)	0.17	−8.0 (−18; 2.4)	0.13	−9.1 (−20; 1.9)	0.11	−5.0 (−17; 6.8)	0.41
Males	556	−4.0 (−8.5; 0.41)	0.075	−5.1 (−10; 0.050)	0.053	−4.5 (−9.9; 0.94)	0.11	−4.7 (−10; 0.85)	0.098
HFpEF	442	−9.5 (−15; −3.6)	**0.0019**	−9.9 (−17; −3.2)	**0.0039**	−9.4 (−16; −2.4)	**0.0091**	−8.9 (−16; −1.7)	**0.015**
Females	191	−11 (−21; −0.96)	**0.033**	−12 (−23; −0.14)	**0.049**	−11 (−23; 1.4)	0.084	n.a.	n.a.
Males	251	−8.6 (−16; −1.2)	**0.024**	−8.3 (−17; 0.0084)	0.051	−7.7 (−17; 1.2)	0.090	−6.0 (−15; 3.2)	0.21
HFpEF borderline	324	−1.9 (−8.7; 4.8)	0.57	−3.0 (−11; 4.7)	0.44	−2.5 (−10; 5.4)	0.53	−3.9 (−12; 4.4)	0.35
Females	82	−11 (−23; 1.4)	0.086	−9.7 (−26; 6.5)	0.24	−12 (−29; 6.3)	0.21	−9.5 (−33; 14)	0.43
Males	242	0.71 (−7.2; 8.6)	0.86	−1.0 (−10; 8.1)	0.83	0.50 (−8.9; 9.9)	0.92	0.092 (−10; 10)	0.99
HFrEF	279	−5.4 (−11; −0.28)	**0.040**	−7.1 (−13; −1.2)	**0.019**	−7.0 (−13; −0.60)	**0.033**	−6.3 (−13; 0.18)	0.058
Females	41	−0.14 (−13; 13)	0.98	−10 (−27; 6.7)	0.25	−8.0 (−26; 10)	0.40	n.a.	n.a.
Males	238	−6.5 (−12; −0.79)	**0.027**	−7.4 (−14; −0.90)	**0.027**	−7.6 (−15; −0.49)	**0.037**	−7.6 (−15; −0.30)	**0.043**

*Multivariable linear regression analysis with platelet count as dependent variable and PTH as independent variable in phenotypes of cardiac function and sex-specific. Results are presented as beta (β)-estimates for change per one standard deviation in PTH. PTH, parathyroid hormone; N, number of individuals; eGFR, estimated glomerular filtration rate; EF, ejection fraction; HFpEF, heart failure with preserved ejection fraction; HFpEF borderline, heart failure with ejection fraction of 41–49%; HFrEF, heart failure with reduced ejection fraction; n.a., not available due to low sample size*.

a *Sex-adjustment only in overall analysis sample;*

b *Vitamin D status was determined by concentrations of Calcifediol and Calcitriol;*

c *In females additionally adjusted for oral contraceptives, hormone replacement therapy and menstrual bleeding. P-value < 0.05 were highlighted in bold*.

## Discussion

PTH and platelet activation have been independently implicated in the pathogenesis of the HF syndrome (15, 22). However, the sex-specific interplay of these factors, as well as their specific relationship in phenotypes of HF, is currently largely unknown. This study demonstrated an important relation between platelet indices and PTH, which varied in phenotypes of cardiac function and particularly in individuals with symptomatic HF. In addition, the present analysis reports on distinct sex-specific differences in HF phenotypes.

Previous studies in individuals with primary hyperparathyroidism and end-stage renal failure patients have shown positive associations between MPV and PTH; however, sex-specific aspects were not addressed (17, 18). Other research has already demonstrated sex-specific differences for MPV in the general population that was also differentially associated with total mortality (14).

In contrast to the findings for MPV and PTH, an inverse association between PTH and platelet count was found in the total sample, which was present in both men and women. The inverse direction of the association between platelet count and PTH compared to MPV is explained by the fact that platelet count and MPV are physiologically inversely related to keep the overall platelet mass stable (23). Similarly, as for the MPV and PTH relation, sex-specific associations observed between platelet count and PTH were distinct for phenotypes of symptomatic HF: within HFpEF individuals, the inverse association was observed more consistent in females, whereas in HFrEF, the inverse association between PTH and platelet count was found in males only.

The etiology of HF differs between males and females regarding prevalence, risk factors, and comorbidities (2), and in part these differences could be explained by the sex-specific hormones, pregnancy, or preeclampsia (24). Also the pathophysiology differs between both sexes, as females tend to suffer more from a “microvascular” disease with vascular stiffness and systemic inflammation, whereas males tend to present with a more “macrovascular” pattern due to comorbidities such as MI or CAD (3, 25). Indeed, the results in the current analysis also differ between both sexes. The associations between MPV or platelet count, and PTH, if found, were with higher effect sizes in females compared to males. Notably, the association was also independent of known female factors influencing the platelet size, such as menstrual bleeding, hormone replacement therapy, and intake of oral contraceptives. Whether endogenous hormone levels influence the association between platelet indices and PTH in the HF syndrome requires further investigation. Genetically determined testosterone levels have been linked with development of HF, predominantly in men, as shown in a recent Mendelian randomization study (26). Post-menopause in women has been associated with an exponential increase in the incidence of HFpEF compared with men of the same age. Estrogen deprivation in post-menopause has been recognized as an important determinant of diastolic dysfunction as estrogen is shown to modulate many regulatory molecular pathways of cardiac diastolic function (27, 28). The present results further support the importance of hormones by showing an important effect of hormone-containing agents on the association between platelet count and PTH in female HF subjects.

The role of PTH according to HF severity has also been reported. A positive correlation between PTH and NYHA class and PTH and NT-proBNP levels as well as an inverse correlation between PTH and LVEF has been reported in different HF studies (8–11).

A positive relation between increasing age and PTH levels has been previously reported, primarily as a response to changes in serum calcium (29). The results from this study showed a weak positive correlation between age and PTH in males with predominantly HFpEF phenotype and in females with predominantly HFrEF phenotype.

In addition, patients with disorders of the parathyroid gland suffered more frequently from arterial hypertension, left ventricular hypertrophy, arrhythmia, and HF (13). Elevated PTH can stimulate cardiac myocyte hypertrophy, dysfunction of endothelium and vasculature, and hypercalcemia and activate aldosterone *via* RAAS (13). However, a community-based study in the Netherlands did not confirm PTH to be associated with a risk of developing HF or predicting new onset of HFpEF or HFrEF (30). Subjects with primary hyperparathyroidism and thus elevated concentrations of PTH presented with higher MPV compared to age- and sex-matched healthy controls (17). Higher MPV could suggest the presence of metabolically and enzymatically hyperactive platelets in HF individuals (17). Activated platelets release a plethora of different proinflammatory mediators that promote immune response, angiogenesis, and fibrosis (31, 32). Hypercalcemia can lead to oxidative stress and inflammation in the heart and finally contribute to cardiomyocyte necrosis (8). However, calcium is required as a cofactor in blood coagulation; a lack of calcium can also impair cardiac function and affect HF progression (33, 34). The presence of PTH-related protein and vitamin D receptors on platelets might lead to platelet activation after direct binding or after PTH-initiated increase of vitamin D or PTH-initiated increase of calcium (18, 19). The described pathways of platelet activation can result in a hypercoagulable state, an already recognized risk factor in HF syndrome (35). Vitamin D has been reported to have anti-inflammatory properties, and given the presence of vitamin D receptors in cardiac myocytes, vitamin D supplementation has been suggested as a possible supporting therapy in HF syndrome (36, 37). Indeed, the VINDICATE study showed the beneficial effects of Vitamin D supplementation on cardiac function and LV structure in patients with chronic HF and vitamin D deficiency for a duration of 1 year (38). On the other hand, suppressing PTH by vitamin D intake might present a potential therapeutic target to prevent PTH-driven endothelial dysfunction, atherosclerosis, and platelet activation as leading causes of cardiac ischemia and HF development (4, 39). In absence of robust experimental evidence for the direct interaction between PTH and platelets, it remains to understand if the observed relation depends on other PTH-dependent mechanisms such as plasma and platelet calcium level and vitamin D concentration and its association with platelet activation. Another hypothesis to be tested for the potential improvement of the clinical outcome of individuals with HF syndrome, based on the present results on the interaction between PTH and platelets, could be the addition of antiplatelet agents in HF patients with higher PTH concentration. To increase the understanding of the interaction between PTH and platelet activation in HF phenotypes, a prospective investigation with specific platelet function tests depicting different aspects of platelet activation, that is, platelet aggregation and platelet procoagulant function, is needed. Furthermore, well-designed randomized controlled trials could importantly inform whether attenuating the levels of PTH intake and/or impeding platelet aggregation and procoagulant function by Vitamin D and antithrombotic agents, respectively, will decrease HF risk or mitigate its progression. Sex-related differences from biological mechanisms to treatment effects and prognosis have been already described in HF patients (40). Our findings for the sex differing association between PTH and platelet indices further support the recommendation to keep the sex-specific focus in future mechanistic, translational, and interventional studies.

## Conclusion

The results of this analysis report important differences for the association between biomarkers of platelets and PTH that vary between sexes and with the phenotype of cardiac dysfunction. These differences are present independent of vitamin D status, CVRFs, and comorbidities. Particularly in phenotypes of symptomatic HF, distinct associations in males and females were observed, suggesting a sex-specific mechanism involved in the interaction between PTH and platelets. Further mechanistic studies are warranted to understand the effect of PTH at the molecular level of platelets, including the role of endogenous hormones in HF phenotypes.

## Data Availability Statement

The original contributions presented in the study are included in the article/Supplementary Material, further inquiries can be directed to the corresponding author/s.

## Ethics Statement

The studies involving human participants were reviewed and approved by Ethics committee University Medical Centre Mainz, reference number 837.319.12 (8420-F). The patients/participants provided their written informed consent to participate in this study.

## Author Contributions

BD, MP-N, and PW designed and performed research and wrote the manuscript. FM, S-OT, and MH contributed to discussion of results and to the critical review of the manuscript. AS performed the statistical analysis. NA, MH, SS-T, JP, TG, HC, KL, and TM contributed in critically reviewing the manuscript. All authors have read, critically reviewed, and approved the manuscript in its current form.

## Conflict of Interest

PW received research funding from Boehringer Ingelheim, Bayer Healthcare, Daiichi Sankyo Europe, Novartis, AstraZeneca, Evonik, Sanofi-Aventis, Bayer Vital, AstraZeneca, DiaSorin and Evonik and received nonfinancial support from DiaSorin and I.E.M. JH. JP reports personal fees from Bayer AG and Boehringer Ingelheim. S-OT has received personal fees from Philips Health Care for a lecture outside the submitted work. The remaining authors declare that the research was conducted in the absence of any commercial or financial relationships that could be construed as a potential conflict of interest.
