# Supplementary material for: Sex-Specific Relationship Between Parathyroid Hormone and Platelet Indices in Phenotypes of Heart Failure—Results From the MyoVasc Study
Source: Front Cardiovasc Med. 2021 Jun 16;8:682521. doi: 10.3389/fcvm.2021.682521 (PMC8245004; doi:10.3389/fcvm.2021.682521)
Supplement: Supplementary file 1 [file Data_Sheet_1.docx]

**Supplement Material**

**Part A. Supplemental Methods**

**Assessment of cardiovascular risk factors (CVRFs) and comorbidities**

Cardiovascular risk factors are determined as:
- Diabetes mellitus was defined as HbA1c ≥ 6.5% or blood glucose level ≥ 126 mg/dL at baseline examination after an overnight fast or at least 8 hours or a blood glucose level of ≥ 200 mg/dL at baseline examination after a fasting period > 5 hours or diagnosed diabetes mellitus by a physician or intake of antidiabetic medication;
- Arterial hypertension was defined as blood pressure ≥140/90 mmHg or diagnose by a physician or intake of antihypertensive medication
- Smoking was interrogated due to active and passive smoking; regular smoking was defined as smoking one cigarette per day or at least seven cigarettes per week or one package per month or one cigarillo per day or at least seven cigarillos per week or two pipes per day
- Dyslipidemia was defined as low density lipoprotein/ high density lipoprotein > 3.5 and/ or triglycerides level > 150 mg/dL or diagnose by a physician intake of lipid modifying medication;
- Obesity was defined as body-mass index (BMI) ≥ 30.0kg/m² or waist-to-hip-ratio > 0,85 in women and >1 in men;
- Family history of myocardial infarction and stroke was defined as myocardial infarction and/or stroke of male first-degree relatives until the age of 60 years or female first-degree relatives until the age of 65 years.

Comorbidities are self-reported and include cardiovascular diseases (CVD), venous thromboembolism (VTE), chronic obstructive pulmonary disease (COPD), cancer and arthritis.

**Assessment of medical treatment**

Calcium and Vitamin D levels are each affected by supplementation and both effect PTH levels; therefore this analysis checked the following medication of the study individuals according to the Anatomical Therapeutic Chemical (ATC) Classification System: Vitamin D supplements (A11CC), calcium supplements (A12A), diuretics (C03), beta-blockers (C07), calcium channel blockers (C08), renin-angiotensin-aldosterone-system antagonists (C09), antiplatelet agents (B01), antilipemic drugs (C10a), antiinflammatory and rheumatic drugs (M01A), glucocorticoids (R03BA), corticosteroids (H02), antibacterial drugs (J01) and immunosuppressant drugs (I04A).

In addition, in females the analysis was adjusted for intake of oral contraceptives, hormone replacement therapy and menstrual bleeding.

**Assessment of cardiac structure and function**

Resting two-dimensional transthoracic echocardiograms were performed according to recommendations by the American and European Societies of Echocardiography using an iE33 echocardiography system (Philips Medical Systems, Amsterdam, The Netherlands) to provide information on chamber dimensions, wall thickness and measures of systolic and diastolic function. The mitral inflow velocity pattern was recorded from the apical four-chamber view with the pulsed waved Doppler sample volume positioned at the tips of the mitral valve leaflets during diastole in expiration. Peak early (E-wave) and late (A-wave) diastolic filling velocities were measured and their ratio (E/A) calculated. The lateral mitral annular early diastolic velocity (E’) was measured by spectral tissue Doppler imaging and the E/E’ ratio determined. Left ventricular ejection fraction (LVEF) was calculated by measurement according to Simpson from the apical four-chamber view. Preserved ejection fraction (PEF) was defined as LVEF≥ 50% and diastolic dysfunction according to one of the following criteria: (E/A< 0.75 and E/E’< 10), (E/A≥ 0.75 and E/E’≥ 10 and DT_E_ ≥ 140ms), or (E/A> 2 and E/E’≥ 10 and DT_E_< 140ms); reduced ejection fraction (REF) was defined as LVEF≤ 40%. The definition of HF phenotypes was further based on a history of HF within the last 12 months and structural or functional heart disease according to ACCF/AHA guideline for the management of heart failure.(1) Patients with a history of HF < 12 months ago or signs and symptoms of HF were classified as ACC/AHA Stage C/D and further categorized by LVEF into HFpEF (LVEF ≥ 50% and diastolic dysfunction), HFpEF borderline (LVEF: 41% to 49%) or HFrEF (LVEF ≤ 40%). Individuals with LVEF ≥ 50% without diastolic dysfunction were categorized as “Stage C/D not classifiable” and excluded for this analysis.

**Part B. Supplemental Tables**

**Table S1. Correlation between PTH and age according to cardiac function in a sex-specific analysis**

|  | **PTH [pg/mL] * age [y]** |
| --- | --- |
|  | Correlation coefficient r |
| Preserved EF | 0.084 |
| - Females | 0.052 |
| - Males | 0.102 |
| Reduced EF | 0.078 |
| - Females | 0.054 |
| - Males | 0.086 |
| HFpEF | 0.088 |
| - Females | -0.032 |
| - Males | 0.159 |
| HFpEF borderline | 0.109 |
| - Females | 0.051 |
| - Males | 0.133 |
| HFrEF | 0.057 |
| - Females | 0.112 |
| - Males | 0.054 |

Pearson's product-moment correlation analysis presenting the correlation between PTH [pg/mL] and age [y] in males and females according to HF phenotypes. Abbreviations: PTH: parathyroid hormone; y: years; EF: ejection fraction; HFpEF: heart failure with preserved ejection fraction; HFpEF borderline: heart failure with ejection fraction of 41-49%; HFrEF: heart failure with reduced ejection fraction.

**References**

1. Yancy CW, Jessup M, Bozkurt B, Butler J, Casey DE, Jr., Drazner MH, et al. 2013 ACCF/AHA guideline for the management of heart failure: a report of the American College of Cardiology Foundation/American Heart Association Task Force on practice guidelines. Circulation. 2013;128(16):e240-327.
